# The chain-mediating role of caregiver burden and illness uncertainty between social support and preparedness among family caregivers of spinal cord injury patients

**DOI:** 10.1371/journal.pone.0351067

**Published:** 2026-06-15

**Authors:** Shuyuan Zhuang, Jiao Wu, Delong Li, li Li, Sihong Dong, Yuantong Zang

**Affiliations:** 1 School of Nursing, Inner Mongolia Medical University, Hohht, China; 2 The Second Affiliated Hospital of Inner Mongolia Medical University, Hohht, China; STIKES Wira Medika PPNI Bali: Sekolah Tinggi Ilmu Kesehatan Wira Medika PPNI Bali, INDONESIA

## Abstract

**Objective:**

This study aimed to examine the effects of social support, caregiver burden, and illness uncertainty on preparedness among family caregivers of individuals with spinal cord injury (SCI), and to analyze the mediating roles of caregiver burden and illness uncertainty in the relationship between social support and preparedness.

**Methods:**

A cross-sectional survey was conducted between June 2024 and April 2025 among conveniently selected SCI patients and their family caregivers from the orthopedics departments of two tertiary hospitals in Hohhot, China. Data were collected using the General Information Questionnaire, the Mishel Uncertainty in Illness Scale, the Social Support Rating Scale, the Chinese version of the Caregiver Burden Inventory, and the Chinese version of the Preparedness for Caregiving Scale.

**Results:**

Correlation analyses indicated that caregivers’ preparedness was positively correlated with social support and negatively correlated with caregiver burden and illness uncertainty. Mediation analyses revealed that social support not only directly enhanced preparedness but also indirectly improved it by alleviating caregiver burden and reducing illness uncertainty. Furthermore, caregiver burden and illness uncertainty were found to form a sequential mediation pathway between social support and preparedness.

**Conclusion:**

Social support enhances preparedness both directly and indirectly through reducing caregiver burden and illness uncertainty. To translate these findings into practice, healthcare providers should routinely assess caregivers’ support needs, integrate burden screening into follow-up visits, and offer uncertainty-management counseling. Hospital- and community-based support programs focusing on skill-building and peer support are also recommended to strengthen caregiver preparedness.

## 1 Introduction

Spinal cord injury (SCI) is a severe neurological condition that leads to motor, sensory, and autonomic dysfunction below the level of injury [1]. Globally, there are approximately 250,000–500,000 new cases of SCI each year [2]. In China, the incidence of traumatic spinal cord injury is increasing, currently estimated at 65.15 per million population [3].McKay et al. [4] demonstrated that, compared with caregivers of patients with other chronic conditions, those caring for individuals with spinal cord injury experience significantly higher risks of physical, psychological, and social morbidities, as well as increased susceptibility to a broad spectrum of illnesses.The researchers argued that because caregivers’ own health and psychological well-being are routinely overlooked, they deserve to be regarded as “invisible patients.”This not only undermines caregivers’ preparedness but may also further affect patient rehabilitation outcomes [5].

Social support, as a modifiable protective factor, has been shown to play an important role in alleviating caregiver burden [6–8]. Previous studies indicate that both illness uncertainty and caregiver burden are negatively correlated with caregiving preparedness [9,10], and illness uncertainty can significantly predict the occurrence of caregiver burden [11]. However, existing research has predominantly focused on pairwise relationships between variables, while the mechanism through which social support influences preparedness via the chain-mediating roles of illness uncertainty and caregiver burden remains insufficiently explored. Unraveling this complex mechanism will deepen our understanding of the relationship between social support and caregiver preparedness. Furthermore, it will provide a theoretical basis for designing multi‑target, continuous psychosocial interventions, thereby systematically improving both preparedness and the overall quality of care.

The ABC-X model, proposed by Reuben Hill in 1949, serves as the theoretical foundation and framework for this study [12]. In this model, “A” denotes the stressor event. In this study, the profound loss of self-care capacity among individuals with spinal cord injury—and their consequent need for extensive assistance—constitutes a significant stressor for family caregivers, manifesting as caregiver burden. “B” represents resources that prevent or buffer crisis and expand the family’s coping repertoire; here, social support functions as the key external resource. “C” signifies the cognitive appraisal of the stressor, that is, the caregiver’s subjective evaluation of how the event will affect them; illness-related uncertainty operationalizes this appraisal. Finally, “X” denotes the outcome of the stress process, which in the present study is caregiver preparedness.The four components of the ABC-X family stress model—stressor, resources, perception, and outcome—can be readily mapped onto the variables examined in this study: caregiver burden, illness uncertainty, caregiving competence, social support, and caregiver preparedness. This theoretical framework supports the proposed hypotheses regarding the influencing factors and pathways affecting preparedness among caregivers of individuals with spinal cord injury.

## 2 Materials and methods

### 2.1 Study participants

A convenience sampling approach was employed to recruit 289 family caregivers of spinal cord injury (SCI) patients from the orthopedic departments of two tertiary hospitals in Inner Mongolia between June 20,2024 and April 30,2025. This study employed a cross-sectional design and convenience sampling. Consequently, the temporality of associations cannot be established, precluding causal inferences, and the use of convenience sampling may introduce selection bias that limits the generalizability of the findings to broader populations.The inclusion criteria for patients were as follows: (1) Diagnosis of SCI confirmed by the International Standards for Neurological Classification of Spinal Cord Injury (ASIA 2011) [13] and corroborated via CT or MRI imaging; (2) Etiology included traumatic or non-traumatic causes with varying degrees of neurological impairment American Spinal Injury Association(ASIA) Impairment Scale grades A/B/C/D; (3) Age ≥ 18 years. The exclusion criteria were as follows: (1) Comorbid dementia or psychiatric disorders, severe organ dysfunction, or systemic illness; (2) Concurrent malignancy. Inclusion criteria for caregivers were as follows: (1) Primary unpaid caregiver (spouse, child, parent, or sibling); (2) Age ≥ 18 years; (3) Provided ≥40 hours of weekly care (including companionship); (4) No cognitive impairment, with intact communication abilities; (5) If multiple caregivers existed, the individual with the longest caregiving duration was selected. Exclusion criteria for caregivers were as follows: Paid caregivers.Approval was obtained from the Institutional Review Board (EFY20240020).The respondents gave informed consent, signed a written informed consent form and volunteered to participate in this study.

#### Sample size calculation.

This study employed structural equation modeling to examine the chain mediation effect. Sample size was estimated a priori using the Satorra–Saris method [14]. The hypothesized model included 4 latent variables and 10 observed indicators, with an estimated 50 degrees of freedom. Parameters were set as follows: RMSEA = 0.08, α = 0.05, power (1-β) = 0.80, yielding a minimum required sample size of 178. Referring to the simulation findings of Fritz & MacKinnon [15] for mediation analysis and the bootstrap stability criteria [16], the required sample size was set at 200. Accounting for a 20% invalid response rate, 300 family caregivers of individuals with spinal cord injury were targeted for recruitment. Bias-corrected percentile bootstrap with 5,000 resamples was applied to test the significance of the chain mediation effects.

### 2.2 Study instruments

#### 2.2.1 General information questionnaire.

The questionnaire was designed by the researcher and comprised two sections. (1) Caregiver’s general information questionnaire, which included age, education level, relationship with the patient, work status, caregiving hours, monthly household income, presence of health insurance, self-rated health status, caregiving experience, and number of co-caregivers. (2) Patient’s general information questionnaire, which included gender, age, ASIA neurological functioning classification, and degree of dependence.

#### 2.2.2 Caregiver Preparedness Scale (CPS).

The Chinese version of the Preparedness for Caregiving Scale, which was adapted for Chinese use by Liu Yanjin [17] and colleagues, was used to assess the readiness of family caregivers of spinal cord injury patients. The scale is unidimensional and comprises eight items: Physical Needs Readiness, Comfort Care Readiness, Developing Service Readiness, Self-Stress Management Readiness, Emotional Needs Readiness, Access to Medical Information Resources Readiness, Response to Emergencies Readiness, and Overall Caregiving Readiness. It is scored on a 5-point Likert scale, with ‘Not at all prepared’ scored as 0, ‘Not well prepared’ scored as 1, ‘Somewhat prepared’ scored as 2, ‘Well prepared’ scored as 3, and ‘Very well prepared’ scored as 4. Scores range from 0 to 32, with higher scores indicating greater preparedness of family caregivers to care for their patients. The Cronbach’s alpha coefficient for this scale was 0.908. Rili Yang’s study [18] explored the applicability of the Chinese version of the CPS among caregivers of patients with spinal cord injury. The results showed that the Chinese version of the CPS had a Cronbach’s α coefficient of 0.909 and a test-retest reliability of 0.877. Confirmatory factor analysis supported its structural validity, indicating that the scale has high applicability among caregivers of spinal cord injury patients in China.Previous studies [19] have examined the psychometric properties and applied utility of the Caregiver Preparedness Scale among family caregivers of individuals with spinal cord injury.The construct validity of the scale was generally good; details are presented in Table 1.

**Table 1:** Results of exploratory and confirmatory factor analyses for the caregiver preparedness scale.

		CPS
EFA	KMO	0.895
	*χ*²	1270.558
	df	28
	*P* value	<0.001
CFA	*χ*²	124.764
	df	20
	pvalue	<0.001
	CFI	0.917
	TLI	0.884
	RMSEA	0.078
	SRMR	0.052
	CR	0.909
	AVE	0.563

CPS, Caregiver readiness.

#### 2.2.3 Caregiver Burden Inventory (ZBI).

The Chinese version of the Zarit Caregiver Burden Scale, which was adapted for Chinese use by Wang Lie et al. [20], was used. It consists of two dimensions: personal burden (12 items) and responsibility burden (6 items), totaling 22 items. The scale is rated on a 5-point Likert scale, with scores ranging from 0 to 4, corresponding to ‘never’ to ‘always’. The total score ranges from 0 to 88, with ≤20 indicating no or mild burden, 21–39 indicating moderate burden, and ≥40 indicating severe burden. The Cronbach’s alpha coefficient for this scale in this study was 0.849.Psychometric properties and application value of caregiver burden scales have been examined in families caring for relatives with spinal cord injury [21].

#### 2.2.4 Family Uncertainty in Illness Scale (MUIS-FM).

The Chinese version of the MUIS-FM, translated and revised by Hongyan Cui [22] based on the original MUIS-FM developed by Mishel, a U.S. scholar, includes four dimensions and a total of 30 items. Of these, 10 items (6, 9, 11, 19, 23, 25, 27, 28, 29, and 30) are reverse-scored, while the remaining items are positively scored. The scale is divided into four dimensions: Ambiguity (11 items), Lack of Clarity (7 items), Lack of Information (4 items), and Unpredictability (3 items). Each item is scored on a 5-point Likert scale, ranging from 1 (strongly disagree) to 5 (strongly agree), with total scores ranging from 30 to 150. Higher scores indicate a greater sense of uncertainty about the disease in the study population. A score exceeding 50% of the total maximum score (75) suggests a high level of uncertainty. The Cronbach’s alpha coefficient for this scale in this study was 0.821.

#### 2.2.5 Social Support Rating Scale (SSRS).

Social support was assessed using the culturally-adapted Social Support Rating Scale (SSRS) developed by Xiao Shuiyuan [23]. This 10-item instrument measures three domains: objective support (3 items), subjective support (4 items), and support utilization (3 items). Total scores reflect the magnitude of perceived social support resources, with higher scores indicating greater support availability. Score interpretation: < 33 (low), 33–45 (moderate), > 45 (high) support. The Cronbach’s alpha coefficient for this scale in this study was 0.75.The psychometric properties and utility of social support scales have been evaluated in family caregivers of persons with spinal cord injury [24].

Exploratory factor analysis was conducted to examine the structural validity of the Social Support Rating Scale (SSRS), the Family Members’ Uncertainty in Illness Scale (MUTS-FM), and the Zarit Caregiver Burden Inventory (ZBI); the results are presented in Table 2.

**Table 2:** Results of exploratory factor analysis for each scale.

		SSRS	MUIS-FM	ZBI
EFA	KMO	0.712	0.832	0.845
	*χ*²	566.772	3084.503	2312.649
	df	45	435	210
	*P* value	<0.001	<0.001	<0.001

MUIS-FM, Illness uncertainty; SSRS, Social support; ZBI, Caregiver Burden; CPS, Caregiver readiness.

### 2.3 Data collection method

A pre-survey was conducted with 30 eligible family caregivers using convenience sampling. The primary aims of this pilot test were to: 1) assess the clarity, comprehensibility, and face validity of the questionnaire items and instructions for the target population, and 2) evaluate the overall feasibility and time burden of the survey procedure. Based on feedback from the pre-survey, the General Information Questionnaire was simplified by removing marital status from the caregiver section and both marital status and length of hospital stay from the patient section to reduce redundancy and improve focus. It is important to note that this pre-survey was not designed for formal psychometric testing; the reliability and validity of the measurement scales were assessed using the data from the main study. The survey was conducted face-to-face on the day of the patient’s discharge from the hospital. Trained investigators and researchers distributed paper questionnaires to the family caregivers, and the estimated completion time was 20–30 minutes. When distributing the questionnaires, a standardized set of instructions was used. The content of the survey and the requirements for completing the questionnaires were explained in detail to the caregivers, and the confidentiality of the information they provided was assured.During data screening, questionnaires with incomplete general information or with over 5% of items missing from the scales were excluded from all analyses. For the retained valid questionnaires, the item-level missing data was minimal (<1% for any scale). Given this pattern, no data imputation was performed, and a pairwise deletion approach was used in the statistical tests. Out of 300 distributed questionnaires, 289 were valid, yielding a response rate of 96.3%.

### 2.4 Statistical analysis

All statistical analyses were performed using SPSS 25.0 (IBM Corp). Categorical variables are presented as frequencies and percentages. Continuous variables with normal distribution were analyzed using independent samples t-tests (two groups) or one-way ANOVA with LSD post-hoc tests (≥3 groups), with results expressed as mean ± standard deviation. Bivariate correlations were assessed using Pearson’s r. Multivariable linear regression analyses employed a stepwise approach.Path analysis was conducted using AMOS 29.0. This study employed the bias-corrected percentile bootstrap method with 5,000 resamples to calculate the 95% confidence intervals for the indirect effects, to examine the chain mediation of illness uncertainty and caregiver burden in the social support-preparedness relationship.Statistical significance was set at α = 0.05 (two-tailed).For the retained valid questionnaires, the item-level missing data was minimal (<1% for any scale). Given this pattern, no data imputation was performed, and a pairwise deletion approach was used in the statistical tests.

## 3 Results

### 3.1 Demographic characteristics of study participants

Among the 289 patients with spinal cord injuries, 196 (67.82%) are male and 93 (32.18%) are female. The age range of the patients is 18–86 years, with a mean age of 57.85 ± 12.45 years. The ASIA injury classification is as follows: grade A, 35 (12.11%); grade B, 91 (31.49%); grade C, 95 (32.87%); and grade D, 68 (23.53%). The age range of the caregivers is 20–67 years, with a mean age of 43.29 ± 9.55 years. For additional information about the caregivers and patients, please refer to Table 3.

**Table 3:** General information on spinal cord injury patients and carers (n = 289).

Variables	Groups	Number	Percentage
			(%)
Monthly income	<2000	15	5.19
	2-3000	49	16.96
	3-4000	90	31.14
	>4000	135	46.71
Education level	Primary School	22	7.61
	Junior high school	99	34.26
	High School or Junior College	92	31.83
	College and above	76	26.3
Relationship with patients	Child	164	56.75
	Spouse	72	24.91
	Parents	13	4.5
	Other relatives	40	13.84
Health status	Fair	63	21.8
	Favourable	226	78.2
Care hours	＜4h/d	32	11.07
	4-8h/d	80	27.68
	8-12h/d	96	33.22
	＞12h/d	81	28.03
Employment status	Unemployed	70	24.22
	In employment	219	75.78
Medical insurance	No	98	33.91
	Yes	191	66.09
Age of carer	≤40	132	45.67
	>40	157	54.33
Care experience	No	220	76.12
	Yes	69	23.88
Co-caregiver	No	56	19.38
	1	129	44.64
	≥2	104	35.99
Patient’s gender	Male	196	67.82
	Female	93	32.18
Patient’s age	<45	41	14.19
	45-60	120	41.52
	≥60	128	44.29
Dependency level	Not at all dependent	63	21.8
	Mildly dependent	88	30.45
	Mostly dependent	108	37.37
	Completely dependent	30	10.38
ASIA classification	D	68	23.53
	C	95	32.87
	B	91	31.49
	A	35	12.11

### 3.2 Correlation analysis of social support, caregiver burden, illness uncertainty, and caregiving preparedness in spinal cord injury patients

Mean scores of caregivers on the Preparedness for Caregiving Scale, Zarit Caregiver Burden Scale, Illness Uncertainty Scale, and Social Support Scale are 15.65 ± 5.28, 43.01 ± 12.32, 80.69 ± 10.81, and 38.30 ± 7.01, respectively. Caregiver readiness is significantly positively correlated with social support (r = 0.410, p < 0.001); caregiver readiness is significantly negatively correlated with illness uncertainty (r = −0.340, p < 0.001); caregiver readiness is significantly negatively correlated with caregiving burden (r = −0.329, p < 0.001); and illness uncertainty is positively correlated with caregiving burden (r = 0.251, p < 0.001). Detailed results are presented in Table 4.

**Table 4:** Correlations between social support, carer burden, illness uncertainty and readiness to care (r-values).

	Social support	Illness uncertainty	Caregiver burden	Caregiver readiness
SSRS	1			
MUIS-FM	−0.240***	1		
ZBI	−0.247***	0.251***	1	
CPS	0.410***	−0.340***	−0.329***	1

MUIS-FM, Illness uncertainty; SSRS, Social support; ZBI, Caregiver Burden; CPS, Caregiver readiness.

***P < 0.001.

### 3.3 Serial mediation analysis of caregiver burden and illness uncertainty in the relationship between social support and caregiving preparedness

**Fig 1:**
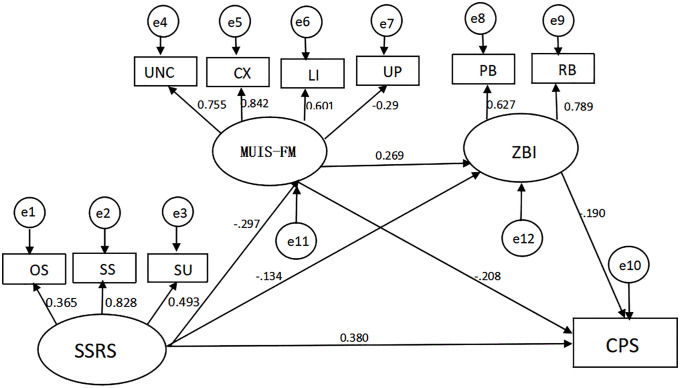
The model path diagram. UNC, uncertainty; CX, complexity; LI, lack of information; UP, unpredictability; PB, personal burden; RB, responsibility burden; OS, objective support; SS, subjective support; SU, support utilization; SSRS, Social support; MUIS-FM, Illness uncertainty; ZBI, Caregiver Burden; CPS, Caregiver readiness.

To balance model complexity with the preservation of multidimensional information, this study used subscale scores as indicators for latent variables. This approach avoids the overparameterization and instability associated with item-level latent variable models, while overcoming the limitations of path analysis based solely on total scale scores, which would obscure the multidimensionality of the constructs (social support, illness uncertainty, and caregiver burden). After developing a mediation model linking illness uncertainty and care burden to social support and caregiving preparedness, we confirm the identifiability of the initial model. The model fit results indicate a chi-square degrees of freedom ratio (*χ*²/df) of 2.107, with GFI = 0.943, IFI = 0.959, TLI = 0.944, and RMSEA = 0.076. While the TLI (0.944) marginally falls short of the stringent 0.95 criterion and the RMSEA (0.076) slightly exceeds the recommended threshold of 0.06, both indices remain within acceptable ranges, suggesting room for further model refinement. The model path diagram is presented in Fig 1, and the corresponding path coefficients are summarized in Table 5.

**Table 5:** Results of chained intermediary analysis.

Path			*β*	B	S.E.	C.R.	*P*
MUIS-FM	<---	SSRS	−0.297	−1.496	0.466	−3.209	0.001
ZBI	<---	MUIS-FM	0.269	0.293	0.096	3.053	0.002
ZBI	<---	SSRS	−0.314	−1.724	0.589	−2.928	0.003
Objective support	<---	SSRS	0.365	1			
Subjective support	<---	SSRS	0.828	4.565	0.969	4.711	<0.001
Support utilisation	<---	SSRS	0.493	1.144	0.245	4.663	<0.001
Uncertainties	<---	MUIS-FM	0.755	1			
sophistication	<---	MUIS-FM	0.842	0.837	0.08	10.507	<0.001
Lack of information	<---	MUIS-FM	0.601	0.33	0.036	9.145	<0.001
Personal burden	<---	ZBI	0.627	1			
Burden of responsibility	<---	ZBI	0.789	0.771	0.138	5.59	<0.001
CPS	<---	MUIS-FM	−0.208	−0.252	0.079	−3.199	0.001
CPS	<---	ZBI	−0.19	−0.211	0.084	−2.503	0.012
CPS	<---	SSRS	0.38	2.322	0.584	3.979	<0.001

MUIS-FM, Illness uncertainty; SSRS, Social support; ZBI, Caregiver Burden; CPS, Caregiver readiness.

This study employed the bias-corrected percentile bootstrap method with 5,000 resamples to calculate the 95% confidence intervals for the indirect effects. The results show that the confidence intervals of indirect effect 1, indirect effect 2, and indirect effect 3 did not contain 0, indicating that the indirect effects of the three paths are significant. These results are presented in Tables 6 and 7. Specifically, social support not only directly and positively predicted caregiver preparedness (effect = 0.380), but also positively influences preparedness through three distinct indirect pathways: by reducing illness uncertainty (indirect effect 1 = 0.062), by alleviating caregiver burden (indirect effect 2 = 0.060), and through a sequential pathway whereby it first reduced illness uncertainty and then mitigated caregiver burden (indirect effect 3 = 0.015). The total indirect effect is 0.137, and the total effect is 0.517, with the mediation accounting for 26.50% of the total effect (0.137/0.517 × 100%).

**Table 6:** Path analysis table for the indirect effect of social support on carer readiness.

Indirect effects1	SSRS - > MUIS-FM - > CPS
Indirect effects2	SSRS - > ZBI - > CPS
Indirect effects3	SSRS - > MUIS-FM - > ZBI - > CPS

**Table 7:** Effect estimates of the impact of social support on carer readiness.

	*β*	95% CI	SE	*P*
Indirect effects1	0.062	(0.023, 0.132)	0.026	0.002
Indirect effects2	0.060	(0.018, 0.140)	0.028	0.005
Indirect effects3	0.015	(0.002, 0.052)	0.011	0.007
Total indirect effect	0.137	(0.070, 0.244)	0.343	<0.001
Direct effect	0.380	(0.241, 0.527)	0.073	<0.001
Total effect	0.517	(0.389, 0.641)	0.064	<0.001

MUIS-FM, Illness uncertainty; SSRS, Social support; ZBI, Caregiver Burden; CPS, Caregiver readiness

## 4 Discussion

### 4.1 Analysis of social support, illness uncertainty, caregiver burden, and caregiving preparedness among family caregivers of spinal cord injury patients

In this study, caregiving preparedness among family caregivers of spinal cord injury patients scored 15.65 ± 5.28, indicating a moderate level of readiness. This may be attributed to several factors: (1) the sudden onset of spinal cord injury often thrusts caregivers into their roles without prior preparation or training, exposing them to multiple stressors and challenges [25]; (2) family caregivers, especially those with patients discharged early, often have insufficient time to acquire caregiving skills and report feeling inadequately prepared; (3) prior research indicates that many caregivers experience considerable psychological and financial strain [26]. Notably, the preparedness score in this study was slightly higher than the 15.45 ± 3.65 reported by Zhu et al. [27], who investigated 305 family caregivers of inpatients with spinal cord injury at a tertiary-grade A specialized rehabilitation hospital in Henan Province, China.This is possibly due to regional differences, healthcare infrastructure, and sample characteristics.

The mean social support score of the studied caregivers was 38.30 ± 7.01, reflecting a moderate level of support. This may indicate the structural characteristics of the current caregiver support system: Confucian family ethics, which emphasize filial piety and spousal unity, have been internalized as a widely accepted norm of family responsibility in Inner Mongolia (specifically manifested in the sample where children and spouses together accounted for over 81.66% of caregivers). This provides caregivers with stable emotional validation and behavioral legitimacy, constituting an important “subjective support” component within social support. However, this family‑centered support model may also objectively limit caregivers’ access to broader social network support (such as community or professional resources), resulting in relatively lower scores in the “objective support” and “utilization of support” dimensions, thereby leading to a moderate overall social support score.

On the other hand, the illness uncertainty score was 80.69 ± 10.81, which, although higher than the norm, remained lower than that observed among caregivers of patients with other conditions such as stroke [28]. We suggest that this finding may be related to the following two factors: First, more than three‑quarters of primary caregivers were employed, and the daily structure and social stability provided by employment may help buffer uncertainty. Second, over half of the caregivers had completed senior secondary education or higher; relatively higher educational attainment likely facilitates more effective acquisition and comprehension of medical information, thereby enhancing their sense of control over the illness trajectory. Notably, the sense of responsibility reinforced by Confucian family ethics not only directly strengthens caregivers’ role adaptation but may also indirectly reduce uncertainty by motivating active health‑related information‑seeking—particularly among those with higher education levels.

The caregiver burden score was 43.01 ± 12.32, slightly lower than the 46.28 ± 12.05 reported in a Nepalese study [29]. Regional medical insurance and pastoral-area policies have cushioned the financial toxicity: the 2022 Inner Mongolia Action Plan for Disability Rehabilitation lists spinal cord injury in the serious-disease insurance segment of the urban–rural resident scheme, raising the reimbursement rate to 75% and adding an immediate “one-stop” settlement [30]. This relative reduction in economic burden explains why the caregiving-burden score in the present study is lower than that reported in comparable Nepalese research.

### 4.2 Relationship between caregiving preparedness and social support, illness uncertainty, and caregiver burden

This study revealed a positive correlation between social support and caregiver preparedness, aligning with findings by Lan Kun et al. [31]. Social support—including emotional, informational, and instrumental help—can enhance preparedness by reducing psychological stress and improving caregiving skills. Culturally, in Inner Mongolia, traditional family and community networks likely strengthen the effect of social support. For instance, clan-based mutual aid and neighborhood support are common, possibly providing more sustained informal assistance than in other regions.

Additionally, a negative correlation was observed between illness uncertainty and caregiver preparedness, consistent with studies on caregivers of traumatic brain injury patients [32]. When caregivers lack clear information about the patient’s prognosis, they may experience anxiety and helplessness, reducing their preparedness. In this regional sample, limited access to specialized healthcare resources and rehabilitation information may exacerbate uncertainty, highlighting a need for culturally and linguistically appropriate educational interventions.

Caregiver preparedness was also negatively correlated with caregiver burden. Wang et al. [33] confirmed that higher burden predicts lower preparedness. In Inner Mongolia, where kinship norms emphasize familial duty, caregivers may experience additional role pressure, intensifying the burden. However, strong family cohesion might also buffer some negative effects—a nuance future studies should explore.

### 4.3 Analysis of the serial mediation effects of illness uncertainty and caregiver burden

The mediation model constructed in this study demonstrates that social support for patients with spinal cord injury has a significant direct impact on caregiver preparedness. This finding is consistent with research findings reported by Susan L. et al. [34]. In this study, social support was identified as a predictor of caregiver preparedness. The following sections discuss specific measures to enhance social support at the family, community, healthcare institution, and societal levels. Healthcare institutions should fully leverage their professional advantages. Before patient discharge, detailed care plans should be developed for caregivers, along with one-on-one care training to ensure they master key skills, such as basic nursing care and prevention of complications [35]. Follow-up systems should be established to regularly assess caregiving situations, promptly address questions encountered by caregivers during the care process, and adjust care plans according to the patient’s rehabilitation progress. Additionally, dedicated consultation hotlines or online service platforms should be established to provide caregivers with convenient access to professional guidance. Communities should establish comprehensive support platforms. On one hand, caregiving skills training courses should be offered, inviting healthcare professionals and rehabilitation therapists to teach caregivers knowledge and skills related to daily care and rehabilitation training for spinal cord injury patients. On the other hand, caregiver support groups should be established, organizing regular gatherings where caregivers can achieve emotional resonance and obtain practical advice through sharing experiences and exchanging insights. Meanwhile, communities should recruit volunteers to provide temporary care, assistance with purchasing daily necessities, and other services, allowing caregivers necessary time for rest and relaxation. The family is the primary source of emotional and practical support for caregivers. Through organizing family meetings, each member’s role in caregiving can be clarified, such as having members with time advantages share daily companionship and assist with rehabilitation training tasks, thereby alleviating the primary caregiver’s pressure. Simultaneously, family members should regularly engage in emotional exchange activities, listen to caregivers’ concerns, provide full understanding and encouragement, and help them release negative emotions [36]. Society as a whole should work together to enhance public awareness and attention towards spinal cord injury patients and their caregivers. Media should promote the hardships and contributions of caregivers through reporting related stories and producing public service announcements, thereby increasing social recognition. By enhancing social support for caregivers of spinal cord injury patients at various levels, caregiver preparedness can be improved.

In the indirect pathway, caregiver burden partially mediates the relationship between social support and caregiver preparedness. Low levels of caregiver burden can effectively enhance the impact of social support, thereby maintaining satisfactory quality of life and caregiver preparedness. These findings are consistent with previous research [37] indicating that excessive caregiver burden significantly reduces caregiver preparedness. Furthermore, illness uncertainty partially mediates the relationship between caregiver social support and caregiver preparedness. The results of this study suggest a negative correlation between social support for caregivers of spinal cord injury patients and their illness uncertainty, which is consistent with previous research findings [38]. Caregivers of spinal cord injury patients frequently encounter sudden and stressful events without adequate preparation and lacking basic information about the condition and its treatment, resulting in insufficient caregiving preparedness. Therefore, reducing caregivers’ illness uncertainty can serve as a key step in increasing social support and enhancing their caregiving preparedness. Additionally, the results of this study indicate that the influence of social support on caregiver preparedness for spinal cord injury patients is realized through a sequential mediating process involving illness uncertainty and caregiver burden. When a family member experiences a spinal cord injury, it imposes a heavy burden on family caregivers, leading to potential uncertainties in patient treatment and rehabilitation, which subsequently affect their internal responses. Illness uncertainty plays a crucial role in this context. Caregivers with higher levels of illness uncertainty may be more likely to experience persistent negative emotions, which could potentially hinder their preparedness. In contrast, caregivers with lower illness uncertainty tend to demonstrate better preparedness for caregiving, which may contribute to improved quality of care and enhanced quality of life for the patient.Therefore, interventions aimed at enhancing caregiver preparedness among family caregivers of individuals with spinal cord injury should concurrently provide clear and ongoing information about the disease and its prognosis to reduce illness uncertainty, and deliver targeted skills training and emotional support to effectively alleviate caregiver burden.

This study employed convenience sampling and recruited primary family caregivers of patients with spinal cord injury from only two tertiary-grade A hospitals in Hohhot; the single-source and geographically concentrated sample may have introduced selection bias, limiting the generalizability of the findings to other healthcare institutions and community populations in Inner Mongolia.To minimize selection bias, future studies should adopt a multistage stratified random sampling design across the regional rehabilitation network, explicitly stratifying by ethnicity, urban/rural residence, and injury severity. Furthermore, establishing a longitudinal cohort across districts, cities, and banners/counties is warranted to dynamically track the stability of the present findings.

## 5 Conclusion

This study investigated the relationships between caregiver preparedness and social support, caregiver burden, and illness uncertainty among 289 caregivers of patients with spinal cord injuries. The findings indicate that illness uncertainty and caregiver burden act as multiple mediators between social support and caregiver preparedness. Based on these results, we recommend that healthcare professionals prioritize strengthening social support—particularly through structured peer‑support networks and psychoeducational interventions—to simultaneously mitigate illness‑related uncertainty and alleviate caregiver burden. Such targeted approaches are likely to enhance preparedness more effectively than general support measures. These findings underscore the need for healthcare systems to formally integrate caregiver-targeted support protocols into clinical practice guidelines, and for nursing education curricula to incorporate dedicated training on assessing and addressing illness uncertainty and burden in caregiver populations.Furthermore, integrating systematic support interventions into standard care pathways—such as pre‑discharge training and routine follow‑up consultations—can help translate improved preparedness into sustained care quality. Future studies could address the suboptimal model fit observed in this study by enlarging the sample size, integrating additional key variables, or utilizing longitudinal data.Moreover, the cross‑sectional nature of this study prevents causal inferences. Future longitudinal research is necessary to establish causality and strengthen the validity of the findings.

## Supporting information

S1 Data Raw data. (XLSX)
